# Involvement of B cells in non-infectious uveitis

**DOI:** 10.1038/cti.2016.2

**Published:** 2016-02-05

**Authors:** Justine R Smith, Andrew J Stempel, Arpita Bharadwaj, Binoy Appukuttan

**Affiliations:** 1 Eye & Vision Health, Flinders University School of Medicine, Adelaide, South Australia, Australia; 2 Vollum Institute, Oregon Health & Science University, Portland, OR, USA

## Abstract

Non-infectious uveitis—or intraocular inflammatory disease—causes substantial visual morbidity and reduced quality of life amongst affected individuals. To date, research of pathogenic mechanisms has largely been focused on processes involving T lymphocyte and/or myeloid leukocyte populations. Involvement of B lymphocytes has received relatively little attention. In contrast, B-cell pathobiology is a major field within general immunological research, and large clinical trials have showed that treatments targeting B cells are highly effective for multiple systemic inflammatory diseases. B cells, including the terminally differentiated plasma cell that produces antibody, are found in the human eye in different forms of non-infectious uveitis; in some cases, these cells outnumber other leukocyte subsets. Recent case reports and small case series suggest that B-cell blockade may be therapeutic for patients with non-infectious uveitis. As well as secretion of antibody, B cells may promote intraocular inflammation by presentation of antigen to T cells, production of multiple inflammatory cytokines and support of T-cell survival. B cells may also perform various immunomodulatory activities within the eye. This translational review summarizes the evidence for B-cell involvement in non-infectious uveitis, and considers the potential contributions of B cells to the development and control of the disease. Manipulations of B cells and/or their products are promising new approaches to the treatment of non-infectious uveitis.

Autoimmune and autoinflammatory uveitis is a heterogeneous group of diseases characterized by non-infectious inflammation within the eye.^1^ Although uveitis is an uncommon medical condition in the general community, it is a leading cause of visual disability and loss of sight,^2^ and it is associated with a marked reduction in the patient's quality of life.^3^ Conventional immunosuppressive drugs, delivered systemically or locally, are not universally effective for uveitis, and side effects further limit their use.^4^ Today considerable research is focused on understanding the basic mechanisms of the inflammation and developing biological approaches that target key pathogenic cells or molecules.

The involvements of helper T lymphocytes and monocytes or macrophages in uveitis have been well established in many studies using experimental models, and through observations made in patients suffering from immune-mediated uveitis.^1, 5, 6^ In contrast, there has been relatively little investigation of the participation of B lymphocytes in the disease. However, scattered across the peer-reviewed literature are reports that implicate B cells in various forms of experimental and clinical uveitis. In addition, B-cell-targeted treatments have recently been used effectively in the clinic for some recalcitrant forms of intraocular inflammation.^7, 8, 9^

This translational review presents the lines of evidence for B-cell involvement in non-infectious uveitis and provides discussion of the potential roles of B cells in the pathogenic and regulatory mechanisms of disease. The review begins with an overview of uveitis, and summaries of B-cell immunology and unique aspects of ocular immunology. In the conclusion to the review, recommendations are made for future directions of study in this field.

## Human non-infectious uveitis and the experimental models

### Definition and classification of uveitis

**Figure 1:**
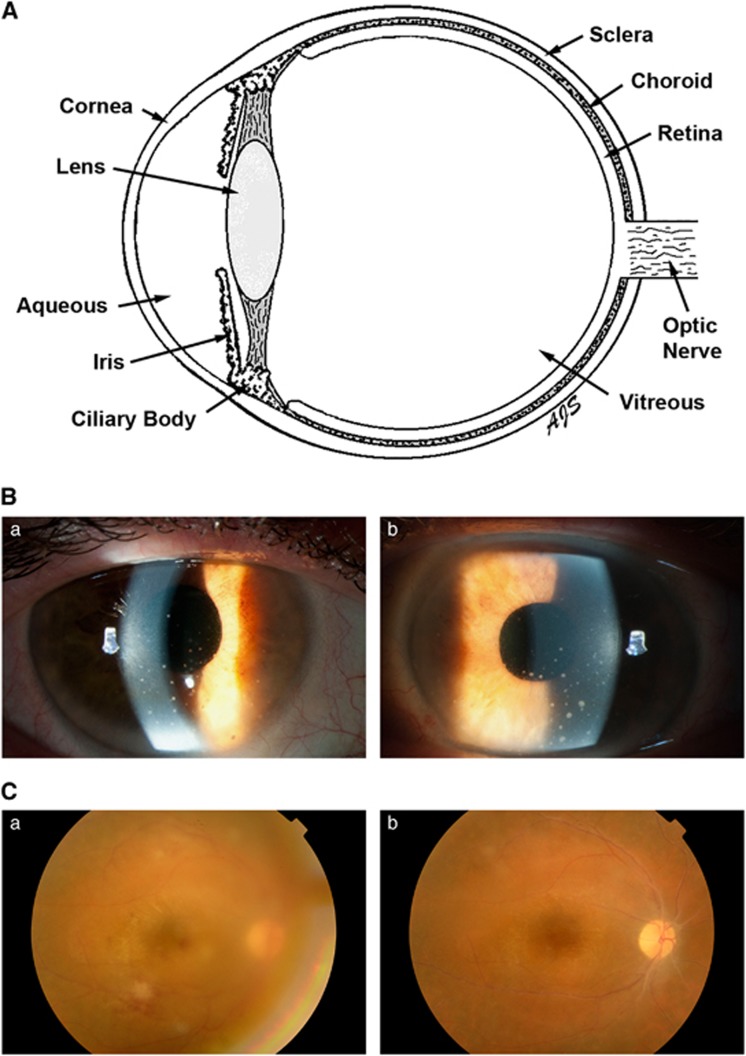
(**A**) Cartoon of the human eye in cross-section. Anterior uveitis is based in the anterior chamber, which is the space bordered by the cornea, the iris and the lens, that is filled with aqueous. Posterior uveitis is based in the retina and/or choroid. Intermediate uveitis is based in the vitreous. (**B**) Clinical photographs of the right (a) and left (b) anterior eyes of a patient with anterior uveitis. Circular whites spots (keratic precipitates), formed by collections of leukocytes, are present on the interior surface of the cornea. (**C**) Clinical photographs taken through the dilated pupil of the right posterior eye in a patient with posterior uveitis. When uveitis is active (a), there are fluffy white patches of retinal inflammation with hemorrhages, and the view is hazy due to secondary inflammation in the vitreous. As the uveitis goes into remission (b), the vitreous inflammation resolves, and there is a crisp view of the retina, which is without whitening or hemorrhages.

Uveitis is a diverse group of inflammatory diseases that involve one or more of the tissues within the eye. By convention,^10^ uveitis is classified anatomically, according to the primary clinical site of the inflammation: ‘anterior uveitis' is based in the anterior chamber; ‘posterior uveitis' is based in the retina or choroid; ‘intermediate uveitis' is based in the vitreous (Figure 1). When the severity of inflammation at two tissue locations cannot be distinguished, these terms are combined (for example, anterior and intermediate uveitis). However, when inflammation is present throughout the eye, with no tissue site predominating, the term ‘panuveitis' is applied.

Inflammation within the eye may be infectious or non-infectious in nature.^1, 11^ Often a specific cause is identified, through consideration of the location of the inflammation, specific ocular signs, clinical course, and patient demographics and co-morbidities. Many infectious agents, including viruses, bacteria, fungi and protozoa, are causes of uveitis. However, infectious uveitis will not be considered further in this review.

Non-infectious uveitis may be the component of a systemic inflammatory disease, or it may be isolated to the eye.^1, 11^ The systemic diseases most often associated with uveitis include: sero-negative HLA-B27-positive spondyloarthropathies; juvenile idiopathic arthritis; sarcoidosis; multiple sclerosis; inflammatory bowel disease; tubulointerstitial nephritis; Behçet disease; and Vogt–Koyanagi–Harada syndrome. Relatively rare, but recently highlighted as inherited syndromes that include non-infectious uveitis, are: familial juvenile systemic granulomatosis and neonatal-onset multisystem inflammatory disease (NOMID).^6^ Systemic vasculitis seldom co-exists with uveitis, although it is commonly associated with scleritis, which is an inflammation of the external scleral coat of the eye.^12^ Diseases confined to the eye may have characteristic clinical pictures. This applies particularly to the ‘white dot' forms of uveitis, including entities such as birdshot retinochoroidopathy and serpiginous choroidopathy, which present with focal inflammation of the retina and/or choroid.^13^

### Current management of non-infectious uveitis

Non-infectious uveitis is often challenging to manage, requiring drugs to suppress the abnormal immune response within the eye.^4^ The first treatment for most patients is corticosteroid, which may be delivered to the anterior eye by eye-drop, but requires either injection around (periocular) or into (intravitreal) the eye, or systemic administration, for intermediate or posterior uveitis. The side effects of corticosteroid generally limit the duration of such treatment. Corticosteroid given systemically has diverse side effects, such as weight gain with cushingoid features and skin striae, psychiatric disturbances, accelerated cardiovascular disease and bone pathology. Locally delivered corticosteroid may precipitate or progress opacification of the lens (cataract) and/or elevate the intraocular pressure, which ultimately may irreversibly damage the optic nerve (glaucoma).

When extended anti-inflammatory intervention is needed, patients are prescribed ‘conventional immunosuppressive drugs'.^4^ Drug classes include anti-metabolites, which disrupt nucleic acid synthesis (for example, methotrexate, azathioprine and mycophenolate mofetil), and calcineurin inhibitors, which reduce proliferation and activation of immune cells (for example, cyclosporine). These drugs achieve control of the inflammation within 12 months in just 30–51% of patients, and to maintain control, many of those individuals require additional treatment with corticosteroid.^14, 15, 16, 17^

Over the past decade, ‘biologic immunosuppressive drugs' that inhibit specific inflammatory mechanisms and that have revolutionized the course of systemic inflammatory diseases, have also been employed to treat non-infectious uveitis. In particular, antibodies that target the inflammatory cytokine, tumor necrosis factor (TNF)-α, are being used with increasing frequency to treat recalcitrant uveitis. The use of antibody-based TNF blockade in uveitis was discussed in a recent expert panel review of 400 publications over 15 years.^18^ In essence, patients with various forms of non-infectious uveitis who fail conventional immunosuppressive drugs frequently show response to this treatment. Challenges include the high cost of the medication, and the potential for life-threatening side effects, including serious infections, new autoimmune disease and malignancy, particularly for uveitis that is confined to the eye. Hypersensitivity reactions and immune-mediated drug resistance may also complicate TNF blockade. Multiple other biologic immunosuppressive drugs with a range of targets (for example, lymphocytes, inflammatory cytokines and co-stimulation) have been used to treat uveitis, but in relatively small groups of patients and/or at relatively few centers.^4^

### Burden of uveitis

Uveitis is a cause of substantial visual morbidity that reduces quality of life and has a high economic cost. The incidence of uveitis is 17–52/100 000 person years in Western nations, and higher in the developing nations.^1^ Uveitis causes vision loss in 70% of individuals who present to a specialist service, with 55% of these persons experiencing legal blindness; and uveitis begins during the working years in up to 90% of patients.^2^ Thus, based on calculations made with US data, the total annual cost of blindness from uveitis equals that of blindness from diabetic eye disease, although uveitis is far less common.^1^ For one in two patients, conventional immunosuppressive drugs will not adequately control the disease and/or treatment-limiting complications will develop.^14, 15, 16, 17^ Quality of life in persons with uveitis is considerably lower than would be expected, based on level of vision alone.^3^

### Experimental models of non-infectious uveitis

The most widely used model of non-infectious uveitis is experimental autoimmune uveoretinitis (EAU), in which a pathological immune response is directed against one of several retinal photoreceptor-associated antigens.^19^ Inflammation is based in the retina, but also involves the anterior chamber and vitreous. The model may be induced in many animals, including non-human primates.^20^ Commonly EAU is induced in the mouse, against interphotoreceptor retinoid-binding protein (IRBP); this was originally performed as a primary immunization, requiring adjuvant, but today adoptive transfer with pathogenic T-cell lines is popular.^5^ Severity of inflammation and structural damage may be graded grossly and histologically.

There are many other experimental models of non-infectious uveitis. Spontaneous uveitis occurs in horses as recurrent equine uveitis,^21^ HLA-A29 transgenic mice^22^ and autoimmune regulator knockout mice.^23^ Intraocular inflammation accompanies some experimental models of systemic inflammation, such as mouse proteoglycan-induced spondylitis^24^ and rat experimental autoimmune encephalomyelitis.^25^ Uveitis may be produced in rodents that are challenged with proteins expressed from transgenes within the retina, and by intraocular injection of inflammatory cytokines or chemokines. Uveitis also may be induced in rodents by intraocular or systemic injection of bacterial products, including lipopolysaccharide and muramyl dipeptide.^26^

## B-cell immunology

### Development of B cells

B cells coordinate the adaptive immune response, alongside T cells. In humans, B cells arise in the bone marrow from CD34^+^ hematopoietic stem cells that are maintained by several populations of stromal cells.^27^ Studies in mice implicate CXCL12-producing reticular cells and osteoblast precursors in this process.^28^ B-cell precursors pass from common lymphoid progenitor, through early B cell, pro-B cell and pre-B-cell stages, as they acquire expression of CD19 and then assemble the B-cell receptor, which is fully functional in the CD34^−^CD10^+^CD19^+^IgM^+^ immature B cell. Immature B cells enter the circulation and travel to spleen, becoming CD10^−^ mature naive B cells, which are capable of specific antigen recognition via their B-cell receptor. Subpopulations of transitional B cells are distinguished in the human on the basis of cell surface markers and functional responses.^29^ Autoreactive B cells are identified for negative selection at the conversions from the pre-B to immature B cell (central tolerance) and transitional stages (peripheral tolerance).^30^

Most mature naive B cells recirculate to secondary lymphoid organs, including lymph nodes, tonsils and mucosa-associated lymphoid tissue, where they enter lymphoid follicles. Here they interact with particulate and soluble antigens, presented in immunological synapses by subcapsular macrophages, dendritic cells and follicular dendritic cells.^31^ Chemokines and cholesterol metabolites direct B-cell movement within the follicle.^32, 33^ CXCR5-expressing mature B cells are directed into the follicle by the chemokine, CXCL13, which is expressed on follicular dendritic cells. CXCL13–CXCR5 signaling also promotes B-cell receptor-mediated B-cell activation.^34^ After internalizing and processing the B-cell receptor and antigen, B cells move to T-cell area under the direction of receptor–ligand interactions involving oxysterol and Epstein–Barr virus-induced G-protein coupled receptor 2, and CCR7 and CCL21, to present antigen to follicular helper T cells and become fully activated.

### Differentiation of B cells

After activation, some B cells secrete specific immunoglobulin M (IgM) as short-lived plasma cells. Other activated B cells move to germinal centers for clonal expansion, and selective somatic hypermutation and class-switch recombination, which enable production of high affinity, class switched antibodies.^35^ These B cells become long-lived CD19^−^ plasma cells or CD27^+^CD19^+^ memory B cells. Shlomchik and Weisel^36^ hypothesize that early germinal center reactions generate memory B cells, and late reactions generate plasmablasts destined become long-lived plasma cells. Memory B cells reside in lymphoid and non-lymphoid organs when not in the circulation.^37^ Long-lived plasma cells are maintained in the bone marrow. According to mouse studies, eosinophils maintain these plasma cells by secreting proliferation-inducing ligand (APRIL) and interleukin (IL)-6.^38^

In addition to conventional B cells, also known as follicular or B-2 B cells, the B lymphocyte population includes innate-like B-1 B cells and marginal zone B cells. These subsets are well described in the mouse, but their existence in humans continues to be discussed. B-1 cells, which develop from common lymphoid progenitors, are characterized by spontaneous secretion of natural antibodies that have broad reactivity against microbial and other danger signals. Rothstein^39^ has taken a functional approach in building a case for a human B cell with this capacity, albeit with different cell surface markers and more specifically directed antibody than characterizes mouse B-1 cells. Marginal zone B cells develop from transitional B cells and are located in the marginal zone of the spleen, for early contact with circulating microbes. Their polyreactive B-cell receptor binds many molecular patterns and they express Toll-like receptors (TLRs) at high levels. Cerutti *et al.*^40^ argue that human marginal zone B cells are not simply memory B cells, as suggested by others.

### Regulatory B cells

A small proportion of B cells have regulatory activities that include production of the immunomodulatory cytokine, IL-10. Rosser and Mauri^41^ provide a comprehensive summary of present understanding of these regulatory B cells, which were first described just over 10 years ago. Regulatory B cells have been equated functionally with regulatory T cells. They may be generated at different stages of B-cell development and differentiation, according to microenvironmental conditions, including activation of TLRs or CD40, or by cytokines. Originally described in mice, regulatory B cells also exist in the human, and diverse involvements have been reported. Iwata *et al.*^42^ identified IL-10-producing B cells in newborns and adults, and in a large group of patients suffering from inflammatory diseases that ranged from rheumatoid arthritis to multiple sclerosis; some patients had expanded numbers of these cells. Flores-Borja *et al.*^43^ showed that human CD19^+^CD24^hi^CD38^hi^ B cells inhibited differentiation of naive CD4^+^ T cells into helper subsets, and promoted differentiation of effector CD4^+^ T cells into regulatory T cells. These activities were curbed in patients with rheumatoid arthritis, who had low numbers of regulatory B cells in blood, but relatively high numbers of such cells in inflamed joints.

## Ocular immunology and inflammation

### Ocular immune privilege

Detection of Ebola virus inside the eye of a physician who survived Ebola virus disease recently focused world interest on the phenomenon of ocular immune privilege.^44^ Beginning in the 1970s, Streilein expanded on the experimental work of Medawar, Billingham and Brent, which had established the concept of the eye as an immune-privileged site;^45^ he worked from the principle that ‘immune-privileged sites allow foreign grafts to survive for extended, often indefinite intervals', and identified microanatomical, cellular and molecular components of ocular immune privilege. This privilege functions to limit inflammation within the eye, where scarring is likely to result in blindness and disadvantage to survival. Thus ocular immune privilege impacts the course of ocular immune responses, and some systemic immune responses

The blood–ocular barriers are microanatomical barriers that restrict the movement of leukocytes into the eye.^46^ The intraocular vascular bed of the anterior eye is located in the iris; tight junctions between iris endothelial cells combined with tight junctions between cells of the posterior iris epithelium and the contiguous non-pigmented ciliary body epithelium form the ‘blood–aqueous barrier'. In the posterior eye, an equivalent ‘blood–retinal barrier' exists as tight junctions between endothelial cells lining the retinal blood vessels and cells of the retinal pigment epithelium. The absence of blood vessels and/or lack of lymphatic drainage of certain ocular surface and intraocular tissues may also limit leukocytic infiltration. However, the extent of a lymphatic-based barrier is unclear as new research suggests a more extensive lymphatic network within the eye than was previously believed.^47^

Deviant immune responses occur when foreign antigens are placed within the eye, in the anterior chamber (anterior chamber-associated immune deviation), vitreous cavity or subretinal space.^48^ This phenomenon has been extensively investigated in the mouse. Spleen-based interactions between ocular F4/80^+^ antigen-presenting cells (believed to be macrophages), B cells and natural killer (NK) T cells and conventional T cells, promote the development of CD8^+^ regulatory T cells that suppress systemic immune responses to antigens previously introduced into the eye.^49, 50^

The eye contains multiple soluble and cell-bound immunomodulatory molecules.^45^ Transforming growth factor (TGF)-β 2 is produced within the eye in latent form, and is converted to active form in the presence of inflammatory cytokines.^51^ This cytokine suppresses activation of infiltrating lymphocytes and macrophages, and confers tolerance-promoting properties on antigen-presenting cells. Other intraocular immunomodulatory cytokines include IL-10, IL-1RA and macrophage migration inhibitory factor. Various neuropeptides—α-melanocyte stimulating hormone, vasoactive intestinal peptide, somatostatin and calcitonin gene-related peptides—also suppress immune responses in the eye.^52^ Constitutive expression of Fas ligand permits the eye to eliminate Fas-bearing infiltrating leukocytes by apoptosis.^53^ Other intraocular immunomodulatory molecules include indoleamine 2,3-dioxygenase, thrombospondin-1 and programmed death-ligand 1. The eye demonstrates low levels of complement activity, along with multiple complement regulatory proteins. Binding of complement C3 fragment iC3b to CR3 on antigen-presenting cells induces the production of TGF-β2.^54^

### Mechanisms of non-infectious uveitis

Non-infectious uveitis is a result of failed ocular immune privilege. In human patients and experimental models, the inflammation is characterized by a mixed intraocular infiltrate of leukocytes.^55, 56^ Studies conducted primarily in mouse EAU indicate a cell-mediated autoimmune response directed against antigens normally confined to the retina, which is coordinated by T cells and macrophages. The obvious human example of autoimmune uveitis is the devastating disease known as sympathetic ophthalmia, in which an injury to one eye is followed by explosive panuveitis in both eyes, as sequestered ocular antigens are suddenly exposed to the systemic immune system.^57^

Experimental autoimmune uveoretinitis is controlled by CD4^+^ helper T cells. Anti-CD4 antibody prevents EAU,^58^ and inflammation is transferred passively by a CD4^+^ T-cell line.^59^ In different forms of EAU, the Th17 subset—characterized by synthesis of IL-17A and other inflammatory cytokines—or the Th1 subset—characterized by synthesis of interferon (IFN)-γ—direct the inflammation. Antibody neutralization of IL-17 limits EAU when B10RIII wild-type mice are immunized with IRBP peptide 161–180 in complete Freund's adjuvant; the same blockade has no effect on EAU induced by infusion of an activated CD4^+^ Th1 cell line that recognizes the same peptide.^60^ These observations suggest that the effector response—Th1 or Th17—depends on the context of antigen presentation. Consistently, ligation of different TLRs on dendritic cells induces different ratios of IL-17:IFN-γ production by co-cultured IRBP-reactive CD4^+^ T cells.^61^ Th1 and Th17 cells may also act together to induce pathology.^62^ Research using transgenic B10RIII mice, which express a T-cell receptor specific to IRBP peptide 161–180 and spontaneously develop EAU, show that IRBP-specific T cells may be activated by non-cognate antigen present in intestinal microbiota.^63^ CD4^+^FoxP3^+^ regulatory T cells expand in the eye during the course of EAU and act to resolve the uveitis.^64^ Regulatory T cells also occur in spleens of mice that have recovered from EAU.^65^

Macrophages are primarily responsible for the tissue destruction that occurs in EAU. Although the retina contains resident macrophages (microglia), newly recruited macrophages effect the destruction of retina by the production of free radicals. This was elegantly demonstrated in independent studies using chimeric mice with bone marrow reconstituted from mice expressing green fluorescent protein (GFP) in their monocyte population; the majority of cells infiltrating the photoreceptor layer at the peak of inflammation were GFP^+^, implying migration from the blood.^66, 67^ Normal retinal structure is retained when early infiltration by macrophages is prevented.^67, 68^ As the severity of EAU peaks, retinal-infiltrating macrophages produce nitric oxide, which is consistent with the M1 macrophage phenotype.^69^

Recently there has been widespread recognition that some forms of non-infectious uveitis are the result of autoinflammation, not autoimmunity.^6^ Innate immune cells are triggered when pathogen-associated or damage-associated molecular patterns interact with extra- or intra-cellular pattern recognition receptors, activating inflammasomes, and the IL-1 family of cytokines.^70^ Clear examples of autoinflammatory uveitis are provided in two hereditary syndromes with gain-of-function mutations: familial juvenile systemic granulomatosis, caused by mutations in the *NOD2* gene; and NOMID, caused by mutations in the *NLRP3* gene. It has been suggested autoinflammation may also contribute to more common forms of uveitis (for example, inflammatory bowel disease-associated uveitis and Behçet uveitis).^6^ Experimental autoinflammatory uveitis may be induced by bacterial lipopolysaccharide or the muramyl dipeptide component of bacterial peptidoglycan.^26^

## B-cell involvement in non-infectious uveitis

In contrast to the intensive research on the roles of T cells and monocytes/macrophages in non-infectious uveitis, there has been little work focused on B-cell involvement in the disease. Most descriptions of B cells and uveitis come from clinical or translational studies involving human ocular tissues or cells. This also contrasts with the situation for T cells and monocytes, for which most work has been conducted in animal models. The evidence for B-cell involvement in non-infectious uveitis includes histopathological and immunocytochemical analyses of intraocular tissues and fluids from patients with uveitis, as well as reports of the therapeutic impact of manipulating B cells (Table 1). The relevance of mouse models to human inflammatory diseases has been actively debated recently.^71, 72^ In relation to B cells in particular, there are substantial differences in B-cell development, phenotypes of differentiated B cells, and immunoglobulin and class switching between man and mouse.^73, 74, 75^

### Human histopathological and immunocytochemical observations

Removal of ocular fluid from eyes of patients with uveitis is a common procedure for diagnostic purposes. Measurement of a high ratio of microbial-specific antibody in ocular fluids versus serum is used routinely to diagnose infectious uveitis.^76^ There are many published reports from different groups that detail the phenotype of leukocytes in aqueous and/or vitreous of patients with various forms of non-infectious uveitis.^55, 77, 78^ B cells are usually present in this exudate, although the proportion ranges widely from being the least common cell to the predominant cell. In contrast to fluid analysis, microscopic evaluations of eye tissue from patients with uveitis are quite uncommon. However, examinations of eyes with forms of uveitis that involve the anterior and/or posterior eye identify B cells in the infiltrate; in some cases these cells outnumber other leukocytes, as described below.

Histopathological assessments conducted over 30 years ago of ocular tissues from four children with uveitis secondary to juvenile idiopathic arthritis consistently described a plasma cell-rich infiltration of the iris and, if examined, the ciliary body.^79, 80, 81^ This impression was recently confirmed by Parikh *et al.*,^82^ who performed a comprehensive immunohistochemical analysis of the whole eye from a boy with juvenile idiopathic arthritis-associated uveitis; CD20^+^ B cells and Ig-expressing plasma cells (primarily IgG, but also IgM and IgA) vastly outnumbered CD3^+^ T cells and CD68^+^ macrophages, leading the authors to conclude that ‘JIA (juvenile idiopathic arthritis)-associated nongranulomatous iridocyclitis is a primarily B-cell-infiltrative process'. Consistent with this observation, vitreous schlieren (optical heterogeneity), detected during ocular surgery in a girl with juvenile idiopathic arthritis-associated uveitis, was the result of high vitreous levels of IgG, IgM and IgA.^83^

In studies dating from the 1980s, tissues from eyes of patients with various forms of posterior uveitis or panuveitis were examined microscopically. In separate reports, The Laboratories of Immunology and Pathology at the National Eye Institute^84, 85, 86^ described immunohistochemical findings in diffuse subretinal fibrosis uveitis syndrome and Vogt–Koyanagi–Harada syndrome. Both diseases were characterized by a predominantly lymphocytic infiltration of the retina and choroid; lymphocytes included CD4^+^ and CD8^+^ T cells and CD22^+^ B cells, but B cells clearly predominated in diffuse subretinal fibrosis uveitis syndrome. B cells, defined on the basis of CD20 expression, were also the most commonly observed lymphocyte in multiple cases of multifocal choroiditis.^87, 88^ The authors of a most remarkable 65-year-long clinicopathological evaluation of 105 eyes with sympathetic ophthalmia reported, ‘Plasma cells are said to be characteristically absent, but 65.0% of steroid-treated and 85.7% of cases before the steroid era showed plasma cell infiltration.'^57^

### Results of studies in experimental models

Although the most common laboratory animal for EAU is the mouse, many other animals are susceptible. Non-human primates develop a disease that is comparable to forms of posterior uveitis syndromes seen in humans, characterized by vitritis, retinitis, retinal vasculitis and chorioretinal lesions.^89^ Fujino *et al.*^20^ described the phenotype of infiltrating leukocytes in 10 *Mucaca mulatta* immunized intradermally with S-antigen emulsified in complete Freund's adjuvant. At 70 days after first immunization, the predominant ocular infiltrating cell was the lymphocyte. For the 7 monkeys that developed clinically apparent uveitis, the proportions of CD22^+^ B cells and CD4^+^ T cells were equal in 3, while in 4 the number of B cells was higher than the number of T cells. In a separate publication,^90^ the same team reported a contrasting picture in mice. B10.A mice were pre-treated with cyclophosphamide and immunized in the footpad and then into the muscle with IRBP in complete Freund's adjuvant, plus *Bordetella pertussis* vaccine. As followed at weekly intervals for 10 weeks after first immunization, EAU was characterized by a macrophage and helper T-cell-rich infiltration, with only rare B cells. B-cell infiltration is also not reported to be a prominent feature of other rodent uveitis models, including lipopolysaccharide-induced uveitis.^26^

### Effect on uveitis of manipulating B cells

A remarkable ‘natural experiment' supports the key role of B cells in uveitis.^91^ A patient with unilateral juvenile idiopathic arthritis-associated uveitis from the age of 3 years, developed severe complications of the inflammation and became legally blind in the affected eye over 14 years despite treatment with conventional systemic immunosuppressive drugs. At the age of 17 years, her uveitis—and the associated joint inflammation—went into remission spontaneously. However, at the same time, she developed recurrent infections and leukocytopenias. Ultimately she was diagnosed with intercurrent common variable immunodeficiency and successfully treated with intravenous immunoglobulin. The basic feature of this immunodeficiency is failure of B-cell differentiation and hypoimmunoglobulinemia. Resolution of the juvenile idiopathic arthritis-associated uveitis after development of common variable immunodeficiency was consistent with significant B-cell involvement in the uveitis. Interestingly, ELISA (enzyme-linked immunosorbent assay) of serum from patients with oligoarticular juvenile idiopathic arthritis detects increased levels of the cytokine, B-cell-activating factor, when uveitis co-exists.^92^

Additional evidence for the participation of B cells in human uveitis comes from results of depleting B cells in patients with uveitis using rituximab. Rituximab is a human–mouse chimeric monoclonal antibody that targets the surface antigen, CD20.^93^ This antigen is first expressed on B cells when they reach the pre-B-cell stage, and expression ceases with differentiation to the plasma cell.^94^ The drug is typically administered in a 2-infusion cycle every 6 months. Various mechanisms of B-cell depletion by rituximab have been described, including antibody-dependent cellular cytotoxicity, complement-dependent cytoxicity and B-cell apoptosis.

Multiple small clinical studies have described therapeutic benefit of treatment with rituximab in patients with different forms of uveitis, including those characterized by B-cell infiltration of the eye. Two retrospective case series^8, 9^ describe the use of rituximab in a total of 18 children with active juvenile idiopathic arthritis-associated uveitis who had failed treatment with TNF blockade; 15 children experienced remission of intraocular inflammation after commencing rituximab, and 13 of these children were able to cease or reduce the dose of prednisolone and/or other systemic immunosuppressive drugs. In a prospective clinical trial involving 20 patients with Behçet disease who were randomized to treatment with rituximab and methotrexate or cyclophosphamide and azathioprine, plus prednisolone, the rituximab and methotrexate group experienced significantly better total adjusted disease index activity at 6 months.^7^ Recalcitrant cases of idiopathic chronic anterior uveitis,^95^ and posterior uveitis caused by the diffuse subretinal fibrosis uveitis syndrome^96^ and Vogt–Koyanagi–Harada disease^97, 98^ also have been treated effectively with rituximab. An unusual case of uveitis associated with autoimmune B-cell-rich lymphocytic hypophysitis entered long-term remission following treatment with the drug.^99^

## Pathogenic and regulatory roles for B cellS in non-infectious uveitis

There are multiple potential mechanisms by which B cells might incite or perpetuate inflammation within the eye (Table 2). Antibody production is one unique characteristic of B cells. Antibody may contribute to inflammation within the eye by activating complement and/or activating innate immune effector cells that are either resident within the eye or recruited during the course of inflammation. B cells may also promote intraocular inflammation by production of inflammatory cytokines, antigen presentation to T cells—which may occur locally—and/or support of T-cell survival. These activities are likely balanced by immunomodulatory mechanisms of ocular immune privilege. New research provides evidence that B cells themselves may exert immunomodulatory effects in uveitis, as regulatory B cells or through effects on transendothelial migration of T cells.

### Antibody-directed mechanisms

In the 1970s and 1980s, there was healthy debate around whether antibodies had a direct pathogenic effect in uveitis.^100^ Elevated levels of serum antibodies to retinal S-antigen were reported in patients with various forms of uveitis, but they were also detected in normal controls and in patients with other eye diseases.^101^ High doses of S-antigen produced a histopathological picture of granulocytic and monocytic infiltration that was consistent with an immune complex disease.^102^ However, there were conflicting reports about the ability of systemic injection of photoreceptor hyperimmune serum to induce EAU in guinea pigs,^100^ and as the important role of CD4^+^ T-cell-mediated pathology in EAU was recognized,^19, 59^ the field moved away from this debate. Yet, the relatively recent description of ‘humanized' EAU in the HLA-DR3 transgenic mice suggests that antibody within the eye is directly inflammatory: adoptively transferred EAU is more severe when serum and cells are transferred, in comparison to transfer of cells alone.^103^

Following classical immunology teaching, antibody within the eye might trigger inflammation by activation of effector leukocytes and/or by activation of complement. These possibilities are discussed below. However, an additional consideration is direct cell toxicity, as occurs in the rare retinal degeneration termed autoimmune retinopathy. Autoimmune retinopathy is characterized by the production of auto-antibodies directed against photoreceptor antigens, such as recoverin and α-enolase.^104^ This may occur as a paraneoplastic syndrome (that is, cancer-associated retinopathy, melanoma-associated retinopathy and bilateral diffuse uveal melanocytic proliferation) or an isolated disease. Retinal degeneration is mediated through antibody-triggered apoptosis of retinal photoreceptors, with sequential activation of caspase 9 and caspase 3, in the absence of a primary inflammatory response.^105^

Antibody complexed with antigen within the eye also might contribute to intraocular inflammation by stimulating effector responses from Fc receptor-bearing leukocytes. Innate effector mechanisms that are triggered when immune complexes bind activating Fc receptors include: mast cell and/or basophil release of vasoactive substances and chemoattractants; neutrophil release of chemoattractants and cytotoxic substances; macrophage oxidative burst, cytotoxicity and release of pro-inflammatory mediators; and antibody-dependent cytotoxicity, effected primarily by NK cells.^106, 107^ Granulocytes, monocytes and NK cells that infiltrate the eye in uveitis, along with resident macrophages and mast cells, could be responsive to immune complexes. Dendritic cells phagocytose antigen–antibody complexes, and present processed antigen to T-cell subsets, including CD4^+^ helper T cells. Classically this interaction takes place in secondary lymphoid organs, but it also may occur within the eye, as discussed below.

Immune complexes trigger complement activation via the classical pathway. Administration of cobra venom, which depletes complement, reduces the severity of EAU induced in guinea pigs with high doses of S-antigen.^108^ Consistently, mice that are genetically deficient in the complement component, C3, develop significantly less severe EAU than wild-type control animals.^109^ Anterior and posterior segments of the human eye exhibit a low level of complement activity, and aqueous of patients with uveitis contains activated complement fragments, C3a, C4a and C5a, at high ratios to total protein, consistent with intraocular complement activation.^110^ Thus the potential exists for intraocular antibody to activate complement and drive inflammation. Indeed, one rationale for the development of the Fab, ranibizumab, for targeting vascular endothelial growth factor in age-related macular degeneration, was the possibility that complement-mediated or cell-dependent cytotoxicity might complicate intravitreal injection of whole antibody.^111^ Fortunately, however, clinical inflammation infrequently complicates the injection of various therapeutic antibodies into the eye for non-uveitis indications, including macular degeneration and vitreoretinal lymphoma.^112, 113^

### Other pro-inflammatory activities of B cells

It is well established that B cells capture antigen via the B-cell receptor, internalize and process this complex, and present antigenic peptide in association with MHC Class II molecule to T cells in secondary lymphoid organs.^31^ Clearly this process might contribute to the pathogenesis of uveitis in patients, with presentation of auto-antigens to helper T cells. As has been studied extensively in relation to multiple sclerosis,^114^ presentation of antigen at the site of inflammation is also reported in the eye.^115^ Fluorescence videomicroscopy was applied to a mouse model of anterior uveitis: labeled T cells from transgenic DO11.10 mice were injected intravenously into BALB/c mice, and the endothelium was activated with lipopolysaccharide. Eyes injected with ovalbumin saw a significantly higher percentage of T cells in contact with antigen-presenting cells than eyes injected with control antigen. In this model, antigen-presenting cells were defined on the basis of labeled ovalbumin uptake and believed to be myeloid-derived. However, it is tempting to speculate the potential for B cells infiltrating the eye in uveitis to also present antigen locally, thereby perpetuating inflammation.

B cells have the capacity to produce inflammatory cytokines. Experiments conducted 15 years ago in the mouse demonstrated conversion of naive B cells to B effector (Be) -1 or -2 cells, with cytokine profiles characteristic of Th1 or Th2 immune responses, when co-cultured with Th1 or Th2 helper T cells, respectively.^116^ Subsequent work has shown human B cells have the capacity to produce inflammatory cytokines, including IFN-γ, IL-17, TNF-α and IL-6.^117, 118, 119^ Studies of cytokine levels in ocular fluids and/or cells from patients with uveitis have identified multiple inflammatory cytokines within the eye, including each of these cytokines; moreover, comparison of cytokines produced in different forms of uveitis suggests that particular profiles may be associated with different uveitis subtypes.^120^ Infiltrating helper T cells and monocytes, and resident macrophages have been considered the primary sources of inflammatory cytokines in uveitis.^5, 56^ However, given that B cells have the capacity to synthesize pro-inflammatory cytokines, it is reasonable to suggest that B cells also contribute to the inflammatory cytokine milieu present in an eye with uveitis.

Independent of antigen presentation, B cells interact with activated CD8^+^ cytotoxic T cells, in part through engagement of CD27 (expressed by B cells) and CD70 (expressed by T cells), to promote survival and proliferation of the T-cell subset.^121^ CD8+ T cells in blood from patients with uveitis express markers of cytotoxicity, including CD107a, and demonstrate enhanced target cell lysis (Hirani S, *et al.* IOVS 2013;54: ARVO E-Abstract 2026). CD8^+^ T cells appear to be particularly important in Behçet uveitis, and have been identified in the aqueous fluid of patients with this disease.^122^

### Immunomodulatory processes

B cells may also have a role in modulating non-infectious uveitis. The involvement of regulatory B cells in mouse uveitis was demonstrated convincingly in a series of experiments published by the Egwuagu Laboratory.^123^ A population of IL-10-producing regulatory B cells, including a subpopulation that also produced IL-35, was generated by exposing splenic B cells to recombinant IL-35. Mice that were genetically defective in IL-35 signaling did not generate these cells and developed more severe EAU, and mice treated with regulatory B cells after induction of EAU experienced substantially less intraocular inflammation than mice treated with IL-10^−^ B cells. The work is yet to be translated to patients with uveitis. However, the group showed that recombinant human IL-35 substantially expanded the population of IL-10-producing B cells in isolates of human peripheral B cells. This population inhibited B cell proliferation induced with phorbol 12-myristate 13-acetate. Thus the authors raised the possibility of using IL-35 or IL-35-producing regulatory B cells to treat uveitis.

Newly described, peptide inhibitor of transendothelial migration (PEPITEM) is a B-cell product that regulates memory T-cell migration across the vascular endothelium during inflammation.^124^ The peptide is produced under the control of adiponectin and binds cadherin 15 on the vascular endothelium; binding results in synthesis of sphingosine-1-phosphate, which in turn binds sphingosine-1-phosphate receptors on T cells as they interact with the endothelium, limiting activation of lymphocyte function-associated antigen-1 and restricting transendothelial migration. This mechanism is compromised in human autoimmune diseases, rheumatoid arthritis and type I diabetes mellitus. Relevance to uveitis was studied in mouse lipopolysaccharide-induced uveitis; treatment of mice with PEPITEM significantly reduced the number of T cells in the intraocular infiltrate.

Clearly immune privilege may impact any inflammatory activities perpetrated by B cells. The Mochizuki Laboratory^125^ have considered this issue, working in the C57BL/6 mouse. In this animal, TGF-β produced by the retinal pigment epithelium limited B-cell proliferation and IgG production by B cells, following stimulation with anti-CD40 antibody, lipopolysaccharide and IL-4. Although the report concentrated on events occurring within the retina, the published data indicated that iris pigment epithelial cells and ciliary body pigment epithelial cells similarly had the capacity to limit B-cell proliferation.

### B-cell entry into the eye

In order for B cells to participate in uveitis, they must first migrate from the circulation into the eye. Leukocytes move from the blood stream into peripheral tissues through complex molecular interactions with the local vascular endothelium.^126^ Tissue-specific combinations of endothelial adhesion molecules and chemokines mediate steps that include tethering, rolling, arrest, firm adhesion and diapedesis. Our group has a long-standing interest in the molecular basis of leukocyte migration across the human retinal vascular endothelium, as occurs in non-infectious posterior uveitis. Endothelial cells may be isolated from human retina dissected from cadaver donor eyes, by a process of enzymatic digestion and selection on the basis of CD31 expression; these cells may be expanded by transduction with lentivirus encoding the human papilloma virus E6/E7 genes.^127^ We have showed that human retinal endothelial cells constitutively express an array of adhesion molecules and chemokines, and the expression of many of these migration signals is increased by inflammatory stimuli.

Recently we have investigated the signals that are used by human B cells for transmigration of retinal endothelium (manuscript under review). We migrated B cell isolates that were prepared from individual healthy adults through transwells populated with human retinal endothelial cells. We examined the effect of antibody blockade of key immunoglobulin superfamily proteins, intercellular adhesion molecule (ICAM)-1, vascular cell adhesion molecule (VCAM)-1 and observed a role for ICAM-1, but not VCAM-1, in B cell transendothelial migration for a majority of the adult subjects. The B cells migrated in significantly higher numbers in the presence of CXCL13, which is a B-cell-selective chemokine that we have detected in human retina. In an unrelated study by another group, aqueous fluid collected from 11 children with juvenile idiopathic arthritis-associated anterior uveitis was found to contain increased soluble ICAM-1, but not VCAM-1, in comparison to control fluid collected from children who did not suffer from uveitis.^128^ This finding suggests ICAM-1 may also be involved in B-cell migration across the iris vascular endothelium, as occurs in anterior uveitis.

## Conclusions

Although basic research in non-infectious uveitis continues to focus on the involvement of various T cell and myeloid cell populations, it is clear that B-cell subsets, including plasma cells, participate in the pathogenesis of this vision-threatening disease. Recent high impact basic publications have highlighted specific roles for B cells in uveitis, and should stimulate interest in clarifying other roles for these cells and their products. For example, re-visiting the question of antibody involvement in uveitis could be informative. Consideration should be given to models in experimental animals other than the mouse, given the apparent lack of major involvement of B cells in mouse EAU. Studies in animal models will need to be translated to the human condition. Although human models are necessarily *in vitro*, it also may be important to use these in elucidating the roles of B cells in uveitis.

In parallel with new basic science studies, recent clinical case series support the possibility of targeting B cells for treatment of uveitis. Treatments that modulate B-cell mechanisms, not B cells directly, will be worthy of consideration, given the roles of B cells not only in promoting, but also in modulating, the process. Options might include targeting B-cell migration into the eye, or augmenting B cell regulatory activities. Clinical trials in humans will ideally progress past non-controlled case series to randomized controlled clinical trials. It may be advisable to initially target specific uveitis syndromes, in which there is strongest evidence for B-cell involvement, such as juvenile idiopathic arthritis-associated uveitis or sympathetic ophthalmia. Such trials will require enrollment across multiple centers, given the low prevalence of uveitis.

Finally, it is worth noting that research on the involvement of B cells in uveitis may have implications for research and development in other ocular diseases. The obvious example is vitreoretinal lymphoma. This highly malignant tumor is almost always of diffuse large B-cell type.^129^ Treatment with intravitreal rituximab, in addition to standard chemotherapeutic drugs, is gaining popularity, but is not a definitive cure for the cancer.^113^ Knowledge of the molecular mechanisms by which B cells access the eye, and the activities of B cells within the eye, is likely to be highly informative in developing more effective treatments for vitreoretinal lymphoma.

## Figures and Tables

**Table 1:** The evidence for involvement of B cells in non-infectious uveitis

*Observations*	*Studies (reference)*
B cells in intraocular leukocytic infiltrate in forms of uveitis including: juvenile idiopathic arthritis-associated uveitis, diffuse subretinal fibrosis uveitis syndrome, Vogt–Koyanagi–Harada syndrome, multifocal choroiditis and sympathetic ophthalmia	57, 79, 80, 81, 82, 84, 85, 86, 87, 88
Schlieren caused by vitreous immunoglobulin in juvenile idiopathic arthritis-associated uveitis	83
Remission of juvenile idiopathic arthritis-associated uveitis coincident with onset of combined variable immunodeficiency	91
Effectiveness of rituximab in forms of uveitis including: juvenile idiopathic arthritis-associated uveitis, Behçet disease, isolated chronic anterior uveitis, diffuse subretinal fibrosis uveitis syndrome, Vogt–Koyanagi–Harada syndrome, autoimmune lymphocytic hypophysitis-associated uveitis	7, 8, 9, 95, 96, 97, 98, 99
Elevated level of B-cell-activating factor in serum of patients with juvenile idiopathic arthritis when uveitis co-exists	92
Prominence of B cells in leukocytic infiltrate of eyes of non-human primates with experimental autoimmune uveoretinitis	20

**Table 2:** Inflammatory and immunomodulatory activities of B cells that may contribute to the development and/or progression of non-infectious uveitis

Inflammatory activities	Processes involving immune complexes
	Activation of innate immune effector cells (for example, granulocytes, monocyte/macrophages, mast cells, NK cells)
	Antigen presentation to CD4+ T cells by dendritic cells
	Complement activation by classical pathway
	Antigen presentation to CD4+ T cells
	Production of inflammatory cytokines (for example, IFN-γ, IL-17, TNF-α, IL-6)
	Promotion of CD8+ T-cell survival and proliferation
Immunomodulatory activities	Production of immunomodulatory cytokines (for example, IL-10, IL-35)
	Regulation of T-cell migration across vascular endothelium

## References

[bib1] de Smet MD, Taylor SR, Bodaghi B, Miserocchi E, Murray PI, Pleyer U et al. Understanding uveitis: the impact of research on visual outcomes. Prog Retin Eye Res 2011; 30: 452–470. 21807112 10.1016/j.preteyeres.2011.06.005

[bib2] Durrani OM, Tehrani NN, Marr JE, Moradi P, Stavrou P, Murray PI. Degree, duration, and causes of visual loss in uveitis. Br J Ophthalmol 2004; 88: 1159–1162. 15317708 10.1136/bjo.2003.037226 PMC1772296

[bib3] Multicenter Uveitis Steroid Treatment Trial Research Group, Kempen JH, Altaweel MM, Holbrook JT, Jabs DA, Sugar EA. The multicenter uveitis steroid treatment trial: rationale, design, and baseline characteristics. Am J Ophthalmol 2010; 149: 550–561 e10. 20097325 10.1016/j.ajo.2009.11.019 PMC2975449

[bib4] Foster CS, Kothari S, Anesi SD, Vitale AT, Chu D, Metzinger JL et al. The Ocular Immunology and Uveitis Foundation preferred practice patterns of uveitis management. Surv Ophthalmol 2015; 61: 1–17. 26164736 10.1016/j.survophthal.2015.07.001

[bib5] Caspi RR. Understanding autoimmune uveitis through animal models. The Friedenwald Lecture. Invest Ophthalmol Vis Sci 2011; 52: 1872–1879. 21450922 10.1167/iovs.10-6909 PMC3101683

[bib6] Willermain F, Rosenbaum JT, Bodaghi B, Rosenzweig HL, Childers S, Behrend T et al. Interplay between innate and adaptive immunity in the development of non-infectious uveitis. Prog Retin Eye Res 2012; 31: 182–194. 22120610 10.1016/j.preteyeres.2011.11.004 PMC3288447

[bib7] Davatchi F, Shams H, Rezaipoor M, Sadeghi-Abdollahi B, Shahram F, Nadji A et al. Rituximab in intractable ocular lesions of Behcet's disease; randomized single-blind control study (pilot study). Int J Rheum Dis 2010; 13: 246–252. 20704622 10.1111/j.1756-185X.2010.01546.x

[bib8] Heiligenhaus A, Miserocchi E, Heinz C, Gerloni V, Kotaniemi K. Treatment of severe uveitis associated with juvenile idiopathic arthritis with anti-CD20 monoclonal antibody (rituximab). Rheumatology (Oxford) 2011; 50: 1390–1394. 21378109 10.1093/rheumatology/ker107

[bib9] Miserocchi E, Pontikaki I, Modorati G, Bandello F, Meroni PL, Gerloni V. Rituximab for uveitis. Ophthalmology 2011; 118: 223–224. 21199719 10.1016/j.ophtha.2010.07.031

[bib10] Jabs DA, Nussenblatt RB, Rosenbaum JT. Standardization of Uveitis Nomenclature Working G. Standardization of uveitis nomenclature for reporting clinical data. Results of the First International Workshop. Am J Ophthalmol 2005; 140: 509–516. 16196117 10.1016/j.ajo.2005.03.057 PMC8935739

[bib11] Jabs DA, Busingye J. Approach to the diagnosis of the uveitides. Am J Ophthalmol 2013; 156: 228–236. 23668682 10.1016/j.ajo.2013.03.027 PMC3720682

[bib12] Rosenbaum JT, Ku J, Ali A, Choi D, Suhler EB. Patients with retinal vasculitis rarely suffer from systemic vasculitis. Semin Arthritis Rheum 2012; 41: 859–865. 22177107 10.1016/j.semarthrit.2011.10.006 PMC4062185

[bib13] Quillen DA, Davis JB, Gottlieb JL, Blodi BA, Callanan DG, Chang TS et al. The white dot syndromes. Am J Ophthalmol 2004; 137: 538–550. 15013878 10.1016/j.ajo.2004.01.053

[bib14] Gangaputra S, Newcomb CW, Liesegang TL, Kacmaz RO, Jabs DA, Levy-Clarke GA et al. Methotrexate for ocular inflammatory diseases. Ophthalmology 2009; 116: 2188–2198 e1. 19748676 10.1016/j.ophtha.2009.04.020 PMC3785935

[bib15] Pasadhika S, Kempen JH, Newcomb CW, Liesegang TL, Pujari SS, Rosenbaum JT et al. Azathioprine for ocular inflammatory diseases. Am J Ophthalmol 2009; 148: 500–509 e2. 19570522 10.1016/j.ajo.2009.05.008 PMC2753718

[bib16] Daniel E, Thorne JE, Newcomb CW, Pujari SS, Kacmaz RO, Levy-Clarke GA et al. Mycophenolate mofetil for ocular inflammation. Am J Ophthalmol 2010; 149: 423–432 e1-2. 20042178 10.1016/j.ajo.2009.09.026 PMC2826576

[bib17] Kacmaz RO, Kempen JH, Newcomb C, Daniel E, Gangaputra S, Nussenblatt RB et al. Cyclosporine for ocular inflammatory diseases. Ophthalmology 2010; 117: 576–584. 20031223 10.1016/j.ophtha.2009.08.010 PMC2830390

[bib18] Levy-Clarke G, Jabs DA, Read RW, Rosenbaum JT, Vitale A, Van Gelder RN. Expert panel recommendations for the use of anti-tumor necrosis factor biologic agents in patients with ocular inflammatory disorders. Ophthalmology 2014; 121: 785–796 e3. 24359625 10.1016/j.ophtha.2013.09.048

[bib19] Caspi RR, Roberge FG, Chan CC, Wiggert B, Chader GJ, Rozenszajn LA et al. A new model of autoimmune disease. Experimental autoimmune uveoretinitis induced in mice with two different retinal antigens. J Immunol 1988; 140: 1490–1495. 3346541

[bib20] Fujino Y, Li Q, Chung H, Hikita N, Nussenblatt RB, Gery I et al. Immunopathology of experimental autoimmune uveoretinitis in primates. Autoimmunity 1992; 13: 303–309. 1472640 10.3109/08916939209112339

[bib21] Gilger BC, Malok E, Cutter KV, Stewart T, Horohov DW, Allen JB. Characterization of T-lymphocytes in the anterior uvea of eyes with chronic equine recurrent uveitis. Vet Immunol Immunopathol 1999; 71: 17–28. 10522783 10.1016/s0165-2427(99)00082-3

[bib22] Szpak Y, Vieville JC, Tabary T, Naud MC, Chopin M, Edelson C et al. Spontaneous retinopathy in HLA-A29 transgenic mice. Proc Natl Acad Sci USA 2001; 98: 2572–2576. 11226280 10.1073/pnas.051595998 PMC30179

[bib23] DeVoss J, Hou Y, Johannes K, Lu W, Liou GI, Rinn J et al. Spontaneous autoimmunity prevented by thymic expression of a single self-antigen. J Exp Med 2006; 203: 2727–2735. 10.1084/jem.20061864 PMC2118158 17116738

[bib24] Rosenzweig HL, Martin TM, Planck SR, Jann MM, Smith JR, Glant TT et al. Anterior uveitis accompanies joint disease in a murine model resembling ankylosing spondylitis. Ophthalmic Res 2008; 40: 189–192. 18421237 10.1159/000119874

[bib25] Adamus G, Amundson D, Vainiene M, Ariail K, Machnicki M, Weinberg A et al. Myelin basic protein specific T-helper cells induce experimental anterior uveitis. J Neurosci Res 1996; 44: 513–518. 8794942 10.1002/(SICI)1097-4547(19960615)44:6<513::AID-JNR1>3.0.CO;2-E

[bib26] Rosenbaum JT, Rosenzweig HL, Smith JR, Martin TM, Planck SR. Uveitis secondary to bacterial products. Ophthalmic Res 2008; 40: 165–168. 18421233 10.1159/000119870

[bib27] LeBien TW. Fates of human B-cell precursors. Blood 2000; 96: 9–23. 10891425

[bib28] Greenbaum A, Hsu YM, Day RB, Schuettpelz LG, Christopher MJ, Borgerding JN et al. CXCL12 in early mesenchymal progenitors is required for haematopoietic stem-cell maintenance. Nature 2013; 495: 227–230. 23434756 10.1038/nature11926 PMC3600148

[bib29] Palanichamy A, Barnard J, Zheng B, Owen T, Quach T, Wei C et al. Novel human transitional B cell populations revealed by B cell depletion therapy. J Immunol 2009; 182: 5982–5993. 19414749 10.4049/jimmunol.0801859 PMC2746373

[bib30] Weill JC, Reynaud CA. The ups and downs of negative (and positive) selection of B cells. J Clin Invest 2015; 125: 3748–3750. 26368305 10.1172/JCI84009 PMC4607130

[bib31] Yuseff MI, Pierobon P, Reversat A, Lennon-Dumenil AM. How B cells capture, process and present antigens: a crucial role for cell polarity. Nat Rev Immunol 2013; 13: 475–486. 23797063 10.1038/nri3469

[bib32] Cyster JG. B cell follicles and antigen encounters of the third kind. Nat Immunol 2010; 11: 989–996. 20959804 10.1038/ni.1946

[bib33] Yi T, Wang X, Kelly LM, An J, Xu Y, Sailer AW et al. Oxysterol gradient generation by lymphoid stromal cells guides activated B cell movement during humoral responses. Immunity 2012; 37: 535–548. 22999953 10.1016/j.immuni.2012.06.015 PMC3465460

[bib34] Saez de Guinoa J, Barrio L, Mellado M, Carrasco YR. CXCL13/CXCR5 signaling enhances BCR-triggered B-cell activation by shaping cell dynamics. Blood 2011; 118: 1560–1569. 21659539 10.1182/blood-2011-01-332106

[bib35] Recaldin T, Fear DJ. Transcription factors regulating B cell fate in the germinal centre. Clin Exp Immunol 2016; 183: 65. 26352785 10.1111/cei.12702 PMC4687514

[bib36] Shlomchik MJ, Weisel F. Germinal center selection and the development of memory B and plasma cells. Immunol Rev 2012; 247: 52–63. 22500831 10.1111/j.1600-065X.2012.01124.x

[bib37] Kurosaki T, Aiba Y, Kometani K, Moriyama S, Takahashi Y. Unique properties of memory B cells of different isotypes. Immunol Rev 2010; 237: 104–116. 20727032 10.1111/j.1600-065X.2010.00939.x

[bib38] Chu VT, Frohlich A, Steinhauser G, Scheel T, Roch T, Fillatreau S et al. Eosinophils are required for the maintenance of plasma cells in the bone marrow. Nat Immunol 2011; 12: 151–159. 21217761 10.1038/ni.1981

[bib39] Rothstein TL, Quach TD. The human counterpart of mouse B-1 cells. Ann N Y Acad Sci 2015; 1362: 143–152. 25988790 10.1111/nyas.12790

[bib40] Cerutti A, Cols M, Puga I. Marginal zone B cells: virtues of innate-like antibody-producing lymphocytes. Nat Rev Immunol 2013; 13: 118–132. 23348416 10.1038/nri3383 PMC3652659

[bib41] Rosser EC, Mauri C. Regulatory B cells: origin, phenotype, and function. Immunity 2015; 42: 607–612. 25902480 10.1016/j.immuni.2015.04.005

[bib42] Iwata Y, Matsushita T, Horikawa M, Dilillo DJ, Yanaba K, Venturi GM et al. Characterization of a rare IL-10-competent B-cell subset in humans that parallels mouse regulatory B10 cells. Blood 2011; 117: 530–541. 20962324 10.1182/blood-2010-07-294249 PMC3031478

[bib43] Flores-Borja F, Bosma A, Ng D, Reddy V, Ehrenstein MR, Isenberg DA et al. CD19+CD24hiCD38hi B cells maintain regulatory T cells while limiting TH1 and TH17 differentiation. Sci Transl Med 2013; 5: 173ra23. 10.1126/scitranslmed.3005407 23427243

[bib44] Varkey JB, Shantha JG, Crozier I, Kraft CS, Lyon GM, Mehta AK et al. Persistence of Ebola virus in ocular fluid during convalescence. N Eng J Med 2015; 372: 2423–2427. 10.1056/NEJMoa1500306 PMC4547451 25950269

[bib45] Streilein JW. Ocular immune privilege: therapeutic opportunities from an experiment of nature. Nat Rev Immunol 2003; 3: 879–889. 14668804 10.1038/nri1224

[bib46] Cunha-Vaz J. The blood-ocular barriers. Surv Ophthalmol 1979; 23: 279–296. 380030 10.1016/0039-6257(79)90158-9

[bib47] Yucel YH, Johnston MG, Ly T, Patel M, Drake B, Gumus E et al. Identification of lymphatics in the ciliary body of the human eye: a novel ‘uveolymphatic' outflow pathway. Exp Eye Res 2009; 89: 810–819. 19729007 10.1016/j.exer.2009.08.010

[bib48] Stein-Streilein J, Streilein JW. Anterior chamber associated immune deviation (ACAID): regulation, biological relevance, and implications for therapy. Int Rev Immunol 2002; 21: 123–152. 12424840 10.1080/08830180212066

[bib49] Lin HH, Faunce DE, Stacey M, Terajewicz A, Nakamura T, Zhang-Hoover J et al. The macrophage F4/80 receptor is required for the induction of antigen-specific efferent regulatory T cells in peripheral tolerance. J Exp Med 2005; 201: 1615–1625. 15883173 10.1084/jem.20042307 PMC2212925

[bib50] Skelsey ME, Mayhew E, Niederkorn JY. CD25+, interleukin-10-producing CD4+ T cells are required for suppressor cell production and immune privilege in the anterior chamber of the eye. Immunology 2003; 110: 18–29. 12941137 10.1046/j.1365-2567.2003.01676.x PMC1783020

[bib51] Cousins SW, McCabe MM, Danielpour D, Streilein JW. Identification of transforming growth factor-beta as an immunosuppressive factor in aqueous humor. Invest Ophthalmol Vis Sci 1991; 32: 2201–2211. 2071334

[bib52] Taylor AW. Ocular immune privilege. Eye 2009; 23: 1885–1889. 19136922 10.1038/eye.2008.382 PMC4698145

[bib53] Griffith TS, Brunner T, Fletcher SM, Green DR, Ferguson TA. Fas ligand-induced apoptosis as a mechanism of immune privilege. Science 1995; 270: 1189–1192. 7502042 10.1126/science.270.5239.1189

[bib54] Sohn JH, Bora PS, Suk HJ, Molina H, Kaplan HJ, Bora NS. Tolerance is dependent on complement C3 fragment iC3b binding to antigen-presenting cells. Nat Med 2003; 9: 206–212. 12514742 10.1038/nm814 PMC1821085

[bib55] Kaplan HJ, Waldrep JC, Nicholson JK, Gordon D. Immunologic analysis of intraocular mononuclear cell infiltrates in uveitis. Arch Ophthalmol 1984; 102: 572–575. 6608345 10.1001/archopht.1984.01040030450024

[bib56] Kerr EC, Copland DA, Dick AD, Nicholson LB. The dynamics of leukocyte infiltration in experimental autoimmune uveoretinitis. Prog Retin Eye Res 2008; 27: 527–535. 18723108 10.1016/j.preteyeres.2008.07.001

[bib57] Lubin JR, Albert DM, Weinstein M. Sixty-five years of sympathetic ophthalmia. A clinicopathologic review of 105 cases (1913—1978). Ophthalmology 1980; 87: 109–121. 7383540 10.1016/s0161-6420(80)35270-6

[bib58] Atalla L, Linker-Israeli M, Steinman L, Rao NA. Inhibition of autoimmune uveitis by anti-CD4 antibody. Invest Ophthalmol Vis Sci 1990; 31: 1264–1270. 1973156

[bib59] Caspi RR, Roberge FG, McAllister CG, el-Saied M, Kuwabara T, Gery I et al. T cell lines mediating experimental autoimmune uveoretinitis (EAU) in the rat. J Immunol 1986; 136: 928–933. 2416842

[bib60] Luger D, Silver PB, Tang J, Cua D, Chen Z, Iwakura Y et al. Either a Th17 or a Th1 effector response can drive autoimmunity: conditions of disease induction affect dominant effector category. J Exp Med 2008; 205: 799–810. 18391061 10.1084/jem.20071258 PMC2292220

[bib61] Peng Y, Han G, Shao H, Wang Y, Kaplan HJ, Sun D. Characterization of IL-17+ interphotoreceptor retinoid-binding protein-specific T cells in experimental autoimmune uveitis. Invest Ophthalmol Vis Sci 2007; 48: 4153–4161. 17724201 10.1167/iovs.07-0251 PMC2567912

[bib62] Wang RX, Yu CR, Mahdi RM, Egwuagu CE. Novel IL27p28/IL12p40 cytokine suppressed experimental autoimmune uveitis by inhibiting autoreactive Th1/Th17 cells and promoting expansion of regulatory T cells. J Biol Chem 2012; 287: 36012–36021. 22936807 10.1074/jbc.M112.390625 PMC3476269

[bib63] Horai R, Zarate-Blades CR, Dillenburg-Pilla P, Chen J, Kielczewski JL, Silver PB et al. Microbiota-dependent activation of an autoreactive T cell receptor provokes autoimmunity in an immunologically privileged site. Immunity 2015; 43: 343–353. 26287682 10.1016/j.immuni.2015.07.014 PMC4544742

[bib64] Silver PB, Horai R, Chen J, Jittayasothorn Y, Chan CC, Villasmil R et al. Retina-specific T regulatory cells bring about resolution and maintain remission of autoimmune uveitis. J Immunol 2015; 194: 3011–3019. 25716996 10.4049/jimmunol.1402650 PMC4459505

[bib65] Kitaichi N, Namba K, Taylor AW. Inducible immune regulation following autoimmune disease in the immune-privileged eye. J Leukoc Biol 2005; 77: 496–502. 15647326 10.1189/jlb.0204114 PMC1363364

[bib66] Kezic J, McMenamin PG. The monocyte chemokine receptor CX3CR1 does not play a significant role in the pathogenesis of experimental autoimmune uveoretinitis. Invest Ophthalmol Vis Sci 2010; 51: 5121–5127. 20463325 10.1167/iovs.10-5325

[bib67] London A, Benhar I, Mattapallil MJ, Mack M, Caspi RR, Schwartz M. Functional macrophage heterogeneity in a mouse model of autoimmune central nervous system pathology. J Immunol 2013; 190: 3570–3578. 23447691 10.4049/jimmunol.1202076 PMC3626074

[bib68] Zhao J, Chen M, Xu H. Experimental autoimmune uveoretinitis (EAU)-related tissue damage and angiogenesis is reduced in CCL2(-)/(-)CX(3)CR1gfp/gfp mice. Invest Ophthalmol Vis Sci 2014; 55: 7572–7582. 25342612 10.1167/iovs.14-15495

[bib69] Robertson MJ, Erwig LP, Liversidge J, Forrester JV, Rees AJ, Dick AD. Retinal microenvironment controls resident and infiltrating macrophage function during uveoretinitis. Invest Ophthalmol Vis Sci 2002; 43: 2250–2257. 12091424

[bib70] Broderick L, De Nardo D, Franklin BS, Hoffman HM, Latz E. The inflammasomes and autoinflammatory syndromes. Annu Rev Pathol 2015; 10: 395–424. 25423351 10.1146/annurev-pathol-012414-040431

[bib71] Seok J, Warren HS, Cuenca AG, Mindrinos MN, Baker HV, Xu W et al. Genomic responses in mouse models poorly mimic human inflammatory diseases. Proc Natl Acad Sci USA 2013; 110: 3507–3512. 23401516 10.1073/pnas.1222878110 PMC3587220

[bib72] Takao K, Miyakawa T. Genomic responses in mouse models greatly mimic human inflammatory diseases. Proc Natl Acad Sci USA 2015; 112: 1167–1172. 25092317 10.1073/pnas.1401965111 PMC4313832

[bib73] Mestas J, Hughes CC. Of mice and not men: differences between mouse and human immunology. J Immunol 2004; 172: 2731–2738. 14978070 10.4049/jimmunol.172.5.2731

[bib74] Garraud O, Borhis G, Badr G, Degrelle S, Pozzetto B, Cognasse F et al. Revisiting the B-cell compartment in mouse and humans: more than one B-cell subset exists in the marginal zone and beyond. BMC Immunol 2012; 13: 63. 23194300 10.1186/1471-2172-13-63 PMC3526508

[bib75] Ichii M, Oritani K, Kanakura Y. Early B lymphocyte development: similarities and differences in human and mouse. World J Stem Cells 2014; 6: 421–431. 25258663 10.4252/wjsc.v6.i4.421 PMC4172670

[bib76] Fekkar A, Bodaghi B, Touafek F, Le Hoang P, Mazier D, Paris L. Comparison of immunoblotting, calculation of the Goldmann-Witmer coefficient, and real-time PCR using aqueous humor samples for diagnosis of ocular toxoplasmosis. J Clin Microbiol 2008; 46: 1965–1967. 18400917 10.1128/JCM.01900-07 PMC2446865

[bib77] Davis JL, Miller DM, Ruiz P. Diagnostic testing of vitrectomy specimens. Am J Ophthalmol 2005; 140: 822–829. 16310459 10.1016/j.ajo.2005.05.032

[bib78] Dick AD, Siepmann K, Dees C, Duncan L, Broderick C, Liversidge J et al. Fas-Fas ligand-mediated apoptosis within aqueous during idiopathic acute anterior uveitis. Invest Ophthalmol Vis Sci 1999; 40: 2258–22567. 10476791

[bib79] Godfrey WA, Lindsley CB, Cuppage FE. Localization of IgM in plasma cells in the iris of a patient with iridocyclitis and juvenile rheumatoid arthritis. Arthritis Rheum 1981; 24: 1195–1198. 7030352 10.1002/art.1780240914

[bib80] Merriam JC, Chylack LT Jr., Albert DM. Early-onset pauciarticular juvenile rheumatoid arthritis. A histopathologic study. Arch Ophthalmol 1983; 101: 1085–1092. 6347150 10.1001/archopht.1983.01040020087015

[bib81] Sabates R, Smith T, Apple D. Ocular histopathology in juvenile rheumatoid arthritis. Ann Ophthalmol 1979; 11: 733–737. 572653

[bib82] Parikh JG, Tawansy KA, Rao NA. Immunohistochemical study of chronic nongranulomatous anterior uveitis in juvenile idiopathic arthritis. Ophthalmology 2008; 115: 1833–1836. 18495247 10.1016/j.ophtha.2008.03.027

[bib83] Nguyen QD, Humphrey RL, Dunn JP, Humayun MS. Elevated vitreous concentration of monoclonal immunoglobulin manifesting as schlieren in juvenile rheumatoid arthritis-associated uveitis. Arch Ophthalmol 2001; 119: 293–296. 11176997

[bib84] Chan CC, Palestine AG, Kuwabara T, Nussenblatt RB. Immunopathologic study of Vogt-Koyanagi-Harada syndrome. Am J Ophthamol 1988; 105: 607–611. 10.1016/0002-9394(88)90052-9 3259837

[bib85] Kim MK, Chan CC, Belfort R Jr., Farah M, Burnier MP, Nussenblatt RB et al. Histopathologic and immunohistopathologic features of subretinal fibrosis and uveitis syndrome. Am J Ophthalmol 1987; 104: 15–23. 3496794 10.1016/0002-9394(87)90287-x

[bib86] Palestine AG, Nussenblatt RB, Chan CC, Hooks JJ, Friedman L, Kuwabara T. Histopathology of the subretinal fibrosis and uveitis syndrome. Ophthalmology 1985; 92: 838–844. 3875819 10.1016/s0161-6420(85)33969-6

[bib87] Dunlop AA, Cree IA, Hague S, Luthert PJ, Lightman S. Multifocal choroiditis: clinicopathologic correlation. Arch Ophthalmol 1998; 116: 801–803. 9639453 10.1001/archopht.116.6.801

[bib88] Shimada H, Yuzawa M, Hirose T, Nakashizuka H, Hattori T, Kazato Y. Pathological findings of multifocal choroiditis with panuveitis and punctate inner choroidopathy. Jpn J Ophthalmol 2008; 52: 282–288. 18773266 10.1007/s10384-008-0566-2

[bib89] Nussenblatt RB, Kuwabara T, de Monasterio FM, Wacker WB. S-antigen uveitis in primates. A new model for human disease. Arch Ophthalmol 1981; 99: 1090–1092. 7236108 10.1001/archopht.1981.03930011090021

[bib90] Chan CC, Caspi RR, Ni M, Leake WC, Wiggert B, Chader GJ et al. Pathology of experimental autoimmune uveoretinitis in mice. J Autoimmun 1990; 3: 247–255. 2397018 10.1016/0896-8411(90)90144-h

[bib91] Amer R, Bamonte G, Forrester JV. Resolution of juvenile idiopathic arthritis-associated uveitis after development of common variable immunodeficiency. Eur J Ophthalmol 2007; 17: 666–668. 17671947 10.1177/112067210701700429

[bib92] Gheita TA, Bassyouni IH, Emad Y, el-Din AM, Abdel-Rasheed E, Hussein H. Elevated BAFF (BLyS) and APRIL in Juvenile idiopathic arthritis patients: relation to clinical manifestations and disease activity. Joint Bone Spine 2012; 79: 285–290. 21733733 10.1016/j.jbspin.2011.05.020

[bib93] Cohen SB. Targeting the B cell in rheumatoid arthritis. Best Prac Res Clin Rheumatol 2010; 24: 553–563. 10.1016/j.berh.2009.11.006 20732652

[bib94] Perosa F, Favoino E, Caragnano MA, Prete M, Dammacco F. CD20: a target antigen for immunotherapy of autoimmune diseases. Autoimmun Rev 2005; 4: 526–531. 16214090 10.1016/j.autrev.2005.04.004

[bib95] Tappeiner C, Heinz C, Specker C, Heiligenhaus A. Rituximab as a treatment option for refractory endogenous anterior uveitis. Ophthalmic Res 2007; 39: 184–186. 17534119 10.1159/000103239

[bib96] Cornish KS, Kuffova L, Forrester JV. Treatment of diffuse subretinal fibrosis uveitis with rituximab. Br J Ophthalmol 2015; 99: 153–154. 24879811 10.1136/bjophthalmol-2013-304686

[bib97] Caso F, Rigante D, Vitale A, Costa L, Bascherini V, Latronico E et al. Long-lasting uveitis remission and hearing loss recovery after rituximab in Vogt-Koyanagi-Harada disease. Clin Rheumatol 2015; 10: 1817–1820. 10.1007/s10067-014-2781-1 25224382

[bib98] Umran RM, Shukur ZY. Rituximab for sight-threatening refractory pediatric Vogt-Koyanagi-Harada disease. Mod Rheumatol 2015, 1–3 in press. 10.3109/14397595.2015.1071234 26154298

[bib99] Xu C, Ricciuti A, Caturegli P, Keene CD, Kargi AY. Autoimmune lymphocytic hypophysitis in association with autoimmune eye disease and sequential treatment with infliximab and rituximab. Pituitary 2015; 18: 441–447. 25224140 10.1007/s11102-014-0592-7

[bib100] Gery I, Mochizuki M, Nussenblatt RB. Retinal specific antigens and immunopathogenic processes they provoke. Prog Retin Res 1986; 5: 75–109.

[bib101] Gregerson DS, Abrahams IW, Thirkill CE. Serum antibody levels of uveitis patients to bovine retinal antigens. Invest Ophthalmol Vis Sci 1981; 21: 669–680. 7298272

[bib102] Rao NA, Wacker WB, Marak GE Jr. Experimental allergic uveitis: clinicopathologic features associated with varying doses of S antigen. Arch Ophthalmol 1979; 97: 1954–1958. 158354 10.1001/archopht.1979.01020020402028

[bib103] Pennesi G, Mattapallil MJ, Sun SH, Avichezer D, Silver PB, Karabekian Z et al. A humanized model of experimental autoimmune uveitis in HLA class II transgenic mice. J Clin Invest 2003; 111: 1171–1180. 12697736 10.1172/JCI15155 PMC152930

[bib104] Grewal DS, Fishman GA, Jampol LM. Autoimmune retinopathy and antiretinal antibodies: a review. Retina 2014; 34: 827–845. 24646664 10.1097/IAE.0000000000000119

[bib105] Shiraga S, Adamus G. Mechanism of CAR syndrome: anti-recoverin antibodies are the inducers of retinal cell apoptotic death via the caspase 9- and caspase 3-dependent pathway. J Neuroimmunol 2002; 132: 72–82. 12417436 10.1016/s0165-5728(02)00314-4

[bib106] Nimmerjahn F, Ravetch JV. Fcgamma receptors as regulators of immune responses. Nat Rev Immunol 2008; 8: 34–47. 18064051 10.1038/nri2206

[bib107] Cerwenka A, Lanier LL. Natural killer cells, viruses and cancer. Nat Rev Immunol 2001; 1: 41–49. 11905813 10.1038/35095564

[bib108] Marak GE, Wacker WB, Rao NA, Jack R, Ward PA. Effects of complement depletion on experimenal allergic uveitis. Ophthalmic Res 1979; 11: 97–107.

[bib109] Read RW, Szalai AJ, Vogt SD, McGwin G, Barnum SR. Genetic deficiency of C3 as well as CNS-targeted expression of the complement inhibitor sCrry ameliorates experimental autoimmune uveoretinitis. Exp Eye Res 2006; 82: 389–394. 16143328 10.1016/j.exer.2005.07.011

[bib110] Mondino BJ, Sumner H. Anaphylatoxin levels in human aqueous humor. Invest Ophthalmol Vis Sci 1986; 27: 1288–1292. 3488296

[bib111] Ferrara N, Damico L, Shams N, Lowman H, Kim R. Development of ranibizumab, an anti-vascular endothelial growth factor antigen binding fragment, as therapy for neovascular age-related macular degeneration. Retina 2006; 26: 859–870. 17031284 10.1097/01.iae.0000242842.14624.e7

[bib112] CATT Research Group, Martin DF, Maguire MG, Ying GS, Grunwald JE, Fine SL et al. Ranibizumab and bevacizumab for neovascular age-related macular degeneration. New Engl J Med 2011; 364: 1897–1908. 21526923 10.1056/NEJMoa1102673 PMC3157322

[bib113] Larkin KL, Saboo US, Comer GM, Forooghian F, Mackensen F, Merrill P et al. Use of intravitreal rituximab for treatment of vitreoretinal lymphoma. Br J Ophthalmol 2014; 98: 99–103. 24158837 10.1136/bjophthalmol-2013-304043

[bib114] Chastain EM, Duncan DS, Rodgers JM, Miller SD. The role of antigen presenting cells in multiple sclerosis. Biochim Biophys Acta 2011; 1812: 265–274. 20637861 10.1016/j.bbadis.2010.07.008 PMC2970677

[bib115] Rosenbaum JT, Ronick MB, Song X, Choi D, Planck SR. T cell-antigen-presenting cell interactions visualized *in vivo* in a model of antigen-specific inflammation. Clin Immunol 2008; 126: 270–276. 18083637 10.1016/j.clim.2007.10.006 PMC2292401

[bib116] Harris DP, Haynes L, Sayles PC, Duso DK, Eaton SM, Lepak NM et al. Reciprocal regulation of polarized cytokine production by effector B and T cells. Nat Immunol 2000; 1: 475–482. 11101868 10.1038/82717

[bib117] Bermejo DA, Jackson SW, Gorosito-Serran M, Acosta-Rodriguez EV, Amezcua-Vesely MC, Sather BD et al. Trypanosoma cruzi trans-sialidase initiates a program independent of the transcription factors RORgammat and Ahr that leads to IL-17 production by activated B cells. Nat Immunol 2013; 14: 514–522. 23563688 10.1038/ni.2569 PMC3631452

[bib118] Duddy ME, Alter A, Bar-Or A. Distinct profiles of human B cell effector cytokines: a role in immune regulation? J Immunol 2004; 172: 3422–3427. 15004141 10.4049/jimmunol.172.6.3422

[bib119] Gagro A, Servis D, Cepika AM, Toellner KM, Grafton G, Taylor DR et al. Type I cytokine profiles of human naive and memory B lymphocytes: a potential for memory cells to impact polarization. Immunology 2006; 118: 66–77. 16630024 10.1111/j.1365-2567.2006.02342.x PMC1782263

[bib120] Ooi KG, Galatowicz G, Calder VL, Lightman SL. Cytokines and chemokines in uveitis: is there a correlation with clinical phenotype? Clin Med Res 2006; 4: 294–309. 17210978 10.3121/cmr.4.4.294 PMC1764804

[bib121] Deola S, Panelli MC, Maric D, Selleri S, Dmitrieva NI, Voss CY et al. Helper B cells promote cytotoxic T cell survival and proliferation independently of antigen presentation through CD27/CD70 interactions. J Immunol 2008; 180: 1362–1372. 18209030 10.4049/jimmunol.180.3.1362

[bib122] Yu HG, Lee DS, Seo JM, Ahn JK, Yu YS, Lee WJ et al. The number of CD8+ T cells and NKT cells increases in the aqueous humor of patients with Behcet's uveitis. Clin Exp Immunol 2004; 137: 437–443. 15270864 10.1111/j.1365-2249.2004.02536.x PMC1809124

[bib123] Wang RX, Yu CR, Dambuza IM, Mahdi RM, Dolinska MB, Sergeev YV et al. Interleukin-35 induces regulatory B cells that suppress autoimmune disease. Nat Med 2014; 20: 633–641. 24743305 10.1038/nm.3554 PMC4048323

[bib124] Chimen M, McGettrick HM, Apta B, Kuravi SJ, Yates CM, Kennedy A et al. Homeostatic regulation of T cell trafficking by a B cell-derived peptide is impaired in autoimmune and chronic inflammatory disease. Nat Med 2015; 21: 467–475. 25894827 10.1038/nm.3842 PMC4425550

[bib125] Sugita S, Horie S, Yamada Y, Mochizuki M. Inhibition of B-cell activation by retinal pigment epithelium. Invest Ophthalmol Vis Sci 2010; 51: 5783–5788. 20538992 10.1167/iovs.09-5098

[bib126] Nourshargh S, Alon R. Leukocyte migration into inflamed tissues. Immunity 2014; 41: 694–707. 25517612 10.1016/j.immuni.2014.10.008

[bib127] Bharadwaj AS, Appukuttan B, Wilmarth PA, Pan Y, Stempel AJ, Chipps TJ et al. Role of the retinal vascular endothelial cell in ocular disease. Prog Retin Eye Res 2013; 32: 102–180. 22982179 10.1016/j.preteyeres.2012.08.004 PMC3679193

[bib128] Sijssens KM, Rijkers GT, Rothova A, Stilma JS, Schellekens PA, de Boer JH. Cytokines, chemokines and soluble adhesion molecules in aqueous humor of children with uveitis. Exp Eye Res 2007; 85: 443–449. 17662277 10.1016/j.exer.2007.06.011

[bib129] Chan CC, Rubenstein JL, Coupland SE, Davis JL, Harbour JW, Johnston PB et al. Primary vitreoretinal lymphoma: a report from an International Primary Central Nervous System Lymphoma Collaborative Group symposium. Oncologist 2011; 16: 1589–1599. 22045784 10.1634/theoncologist.2011-0210 PMC3233294

